# Value of ultrasound fusion imaging in detecting vascular cerebral white matter pathology

**DOI:** 10.1186/s13089-022-00275-5

**Published:** 2022-06-17

**Authors:** Cornelia Brunner, Stephan Joachim Schreiber, Martin Bokemeyer, Gerhard Ransmayr, Walter Struhal, Elisabeth Daniela Olbert, Naela Alhani, Milan Rastislav Vosko

**Affiliations:** 1 Department of Neurology, Karl Landsteiner University of Health Sciences, University Hospital Tulln, Alter Ziegelweg 10, 3430 Tulln, Austria; 2 Department of Neurology, Kepler University Hospital, 2, Krankenhausstraße 09, 4020 Linz, Austria; 3 Department of Neurology, Asklepios Clinic Brandenburg, Anton-Saefkow-Allee 2, 14772 Brandenburg an der Havel, Germany; 4 Department of Neurology, Oberhavel Kliniken, Clinic Hennigsdorf, Marwitzer Straße 91, 16761 Hennigsdorf, Germany

**Keywords:** Ultrasound fusion-imaging, White matter hyperintensities, Transcranial sonography, Magnetic resonance imaging

## Abstract

**Background:**

Transcranial sonography is beside magnetic resonance imaging (MRI) and computed tomography, a well-established imaging method for evaluation of brain parenchyma and already implicated in various neurological disorders as bed-side investigation possibility in clinical routine. The aim of this study was the qualitative assessment detecting vascular white matter hyperintensities (WMHs), with ultrasound fusion-imaging technique (UFI) and to find the optimal location for their visualization in accordance to the grade of WMHs and to possibly providing a standardized protocol for clinical use.

**Results:**

29 patients with WMHs of variable degree quantified according to Fazekas grading scale *(n* = 13 I; *n* = 9 II; *n* = 7 III) and 11 subjects with normal findings on MRI were identified for further analysis. Ultrasound images were analyzed to a standardized protocol and predefined anatomical landmarks. UFI could visualize the MRI-verified WMHs in 147 of 161 localizations (91%). The overall ultrasound detection rate of WMHs increased with higher degree of WMHs burden (I:85%, II:94%, III:97%). The highest sensitivity was achieved at the contralateral central part (CPc) (97%) of the lateral ventricle. The inter-rater analysis between 2 independent raters, who were blinded to the patient’s diagnosis and assessed only the B-mode ultrasound images, indicated an 86% agreement with an overall moderate strength of agreement (*κ*: 0.489, *p* < 0.0005) for all localizations. The highest accordance within raters was shown at the CPc; 92% (*κ*: 0.645, *p* < 0.0005).

**Conclusions:**

This explorative study describes prospectively the ultrasound detection of periventricular vascular WMHs based on MRI lesions using UFI. Transcranial ultrasound (TCS) could serve as an additional screening opportunity for the detection of incidental WMLs during routine TCS investigations to initiate early vascular risk factor modification in primary prevention.

## Background

Cerebral small vessel disease (CSVD) is a common neurological disease and comprises clinical, histopathological and imaging features. It causes about 20% of all strokes worldwide and is related to up to 45% of dementias, late-life depression, behavioral and gait disturbances [1, 2]. The diagnosis of CSVD relies on imaging findings and include white matter hyperintensities (WMHs), small subcortical infarcts, cerebral microbleeds, lacunar infarcts and brain atrophy. WMHs are variable in size and are mainly located periventricular, the deep and subcortical white matter. Pathophysiologically, WMHs have been linked to different degrees of demyelination, axon loss, gliosis, perivascular tissue changes, thickened vessel walls, venous collagenosis or perivascular edema due to intermittent disruption of the blood–brain barrier [3–5].

Less is known about the causal association between CSVD and its clinical sequelae [6]. For instance, vascular cognitive impairment (VCI) is the second cause of cognitive decline which results from different etiologies, like large or strategic stroke, multi-infarct strokes, border-zone infarcts or cerebral small vessel disease. The cause of microstructural changes in VCI are not completely clear. Evidence suggests oxidative stress, neuroinflammation, endothelial dysfunction, neurotransmitter imbalance. There is growing evidence exploring VCI, especially in defining strategies for diagnosis in very early stages or to find specific markers for patients at risk to further develop strategies for accurate diagnosis and preventive patient-tailored therapeutical options [7].

The standard imaging techniques to visualize white matter lesions (WMLs) in CSVD are brain magnetic resonance imaging (MRI) and computed tomography (CT). The Fazekas grading (Grade I–III) scale is the most commonly used classification system to quantify vascular WMLs on brain MRI or CT radiologically [8].

Another, but not routinely applied method of brain parenchyma imaging is ultrasound (US). Nowadays, high-end ultrasound systems achieve high image resolution. Transcranial sonography of brain parenchyma is well established in diagnosing and monitoring degenerative brain disorders [9, 10]. Particularly, transcranial ultrasound assessment of the substantia nigra in neurodegenerative disorders can help to differentiate between Parkinson disease and essential tremor with a sensitivity of  > 90% and a specificity of  > 89% [11]. The usage of TCS also in emergency department setting is a promising application, since it might be able to distinguish, e.g., unclear neurological movement disorders with acute onset in patients presenting at ED [12].

Further with TCS it is feasible to control the position of deep brain stimulation electrodes postoperatively and it is a reliable method for bed-side monitoring of midline shift in stroke and intracranial hemorrhage [13–15].

Ultrasound fusion imaging (UFI) enables the combination of real-time benefits resulting from US and the anatomical clarity of CT/MR images which helps understanding the sometimes difficult interpretation of transcranial US images and thus allows real-time navigation, exact assessment and correlation of specific structures. However, it is highly operator and experience-dependent procedure [16]. In CSVD this application has not been investigated until now. US is noninvasive, non-radiative, easily repeatable at bedside and widely available.

The aim of this study was that with ultrasound fusion imaging technique based on MR images, it is possible to detect vascular WMLs due to their alteration of echogenicity of brain parenchyma with TCS and to examine the optimal localizations for their detection.

## Methods

### Patients

Thirty-six consecutively admitted patients with vascular WMLs on MRI and 12 consecutively admitted subjects with normal MRI brain findings as control group were screened for the study. Additional inclusion criteria were: age > 18 years, suitable temporal bone window and absent contraindication for MRI and UFI. Patients with any acute brain lesions, known neurodegenerative or neuroinflammatory diseases were not included.

We performed this pilot study at the Department of Neurology (location temporarily anonymized). Demographic data, relevant cardiovascular comorbidities and medications were documented (Table 1).

**Table 1:** Demographic and comorbidity characteristics of 40 subjects

	Controls (*n* = 11)	Patients (*n* = 29)	*p *value
Sex			
Male	7 (63.6%)	19 (65.5%)	NS
Female	4 (36.4%)	10 (34.5%)	NS
Age, mean ± SD (years)	49.73 ± 16.33	71.28 ± 9.53	NS
Body mass index ± SD (kg/m^2^)	26.68 ± 3.83	26.05 ± 4.04	NS
Smoking	5 (45.5%)	7 (24.1%)	0.2535
Hypertension	4 (36%)	22 (75.9%)	0.0292
Diabetes mellitus Type II	–	8 (27.6%)	0.0803
Hyperlipidemia	3 (27.3%)	12 (41.8%)	0.4861
Alcohol	–	1 (3.4%)	NS
Silent lacunar infarcts	1 (9.1%)	8 (27.6%)	0.3994
WMLs, Fazekas °I	–	13 (44.8%)	–
WMLs, Fazekas °II	–	9 (31%)	–
WMLs, Fazekas °III	–	7 (24.2%)	–

Data are presented as mean ± standard deviation (SD) or as percentage (%)

*WMLs* white matter lesions

### Ultrasound fusion imaging

All ultrasound investigations were performed by one experienced investigator (temporarily anonymized) with an ESAOTE Mylab Twice Virtual Navigator^®^ ultrasound system (Genoa, Italy) The ultrasound system was equipped with the Virtual Navigation option (Med-Com GmbH, Darmstadt, Germany).

Brain tissue was visualized bilaterally from transtemporal approach using a phased-array transducer (Esaote, PA240, Genoa, Italy) with an operating frequency of 4.0–1.0 MHz in B-Mode followed by fusion of imported MR images in DICOM format. MR images were performed with 1.5 Tesla scanner with axial slices, slice thickness 5.00 mm and 10% gap (GE Medical System Signa HDxt, 1.5 T, Chicago, USA). Only subjects with a suitable transtemporal bone window were included in our study. This was defined as clear visualization of contralateral skull bone and midbrain structures in axial midbrain imaging plane and lateral ventricles as well in cella media plane [17].

The US registration procedure followed a protocol already published in 2015 [18]. For fusion registration procedure TOF sequences and for online analysis during US session FLAIR sequences were used. Grading of WMLs on MRI was carried out by an experienced neuroradiologist (temporarily anonymized) using the Fazekas rating scale for cerebral WML; Fazekas 0 = None or a single punctate WMH lesion, Fazekas I (mild): multiple punctate lesions, Fazekas II (moderate): beginning confluence of lesions (bridging), Fazekas III (severe): large confluent lesions [8].

**Fig. 1:**
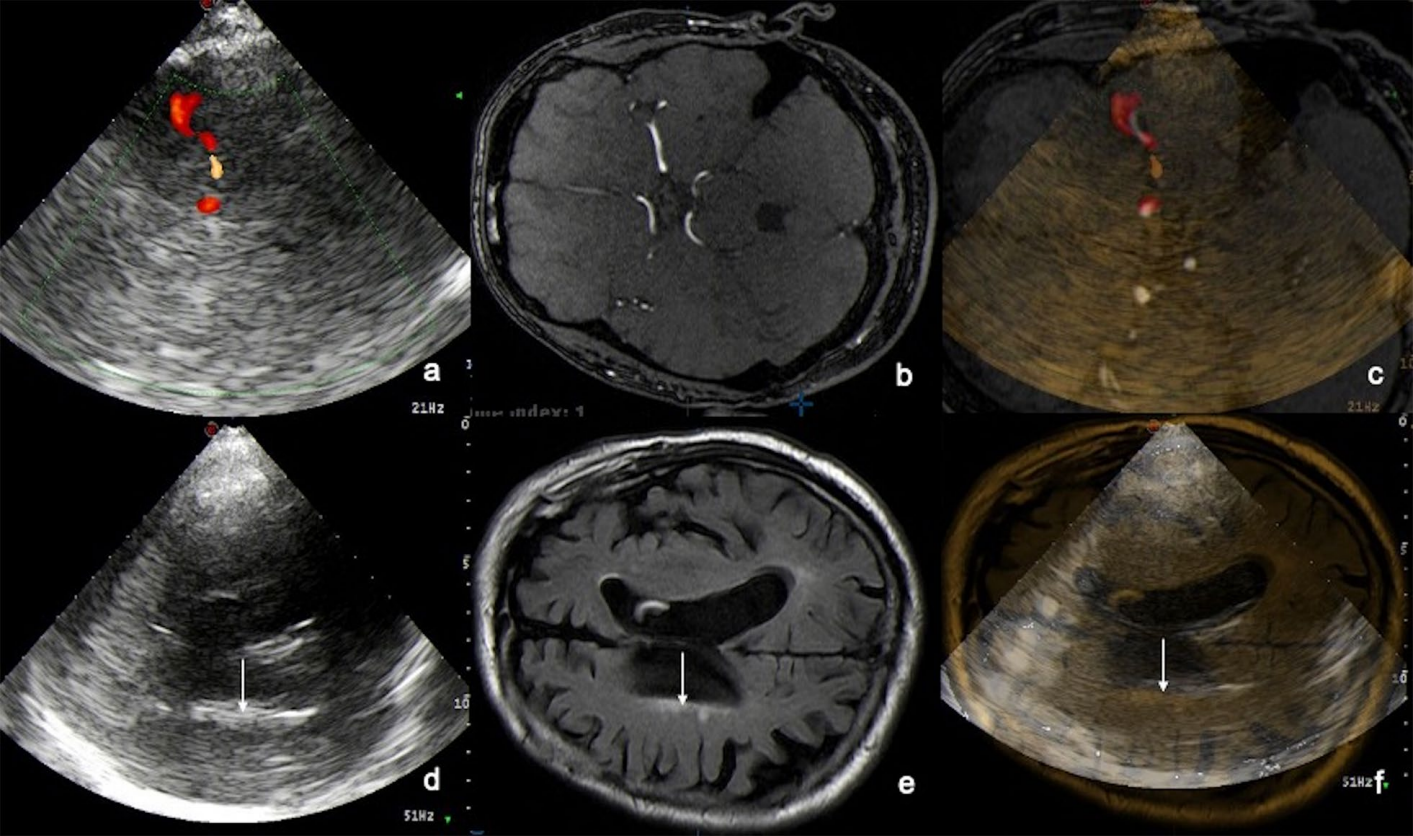
Fusion procedure of ultrasound (US) and MR images. The ipsilateral middle cerebral artery (MCA) is determined as anatomical landmark. After US visualization of the ipsilateral MCA in color-coded duplex ultrasonography (TCCS), axial scanning approach, US images are fused with the corresponding MR images (TOF-sequence) and corrected, if necessary, to match perfectly (**a–c**). During UFI investigation MR FLAIR images are used for online examination (**d**–**f**). **a–c**: Left: TCCS MCA ipsilateral. Middle: MR TOF angiography. Right: Fused images. **d–f**: Left: TCS in B-Mode. Middle: cerebral MR FLAIR image, axial plane. Right: fused images US and MRI. White matter lesions (WMLs) II, Anterior horn contralateral. White arrows indicate the WMLs

During clinical US examination, US and MR images were fused. As anatomical landmark for fusion, we determined a minimum of insonation-length of 1 cm of the ipsilateral middle cerebral artery (MCA) in color Doppler mode in axial scanning approach (Fig. 1). After US visualization of the MCA, US images were fused with the corresponding MRI images (TOF) and the position of the images were corrected to match accurately if necessary (Fig. 1).

According to the main locations of periventricular and deep white matter vascular WMLs, we predefined three anatomical regions of the lateral ventricle for investigation: (a) frontal horn area (FH), dorsal horn area (DH) and the central part area including the deep white matter (CP).

All localizations were examined ipsi- and contra-laterally from each side.

The cella media plane displayed the optimal insonation angle for visualization of the central part of the lateral ventricle bilaterally. The cella media plane was reached by tilting the probe further upward from the midbrain plane axially. Investigation of the frontal, the dorsal horns and the deep white matter was performed in thalamic plane.

### Image analysis

The TCS images in B-mode and all fused images firstly were analyzed from principal investigator online during UFI session. Observations were documented and the images saved in DICOM format. After the first analysis was done, two independent experienced ultra-sonographers (temporarily anonymized), who had been standardized instructed with representative US patterns and were blinded to patients’ diagnosis, evaluated solely B-mode US images for inter-rater analysis. To reduce/exclude the analysis bias the frozen US images selected for further analysis were strictly in accordance with clear and reproducible anatomical landmarks visualized with ultrasound, independent of WMLs load seen on MRI.

### Statistical analysis

The statistical analysis was performed using the SSPS statistical software system (IBM SPSS Statistics for Mac, Version 26.0.0.1, IBM Corp. Armonk, NY, 2019). Patients’ demographic and clinical characteristics are given as means ± standard deviations (SD), as medians and ranges or as percentages. For between-group comparisons the Student´s *t*-test was used, and for categorical variables the Chi-squared test. Fleiss` kappa analysis was performed to assess inter-rater agreement for three raters. Kappa (*κ*) values of < 0 were assumed as poor; 0.01–0.20 as slight; 0.21–0.40 as fair; 0.41–0.60 as moderate; 0.61–0.80 as substantial and 0.81–1.00 as almost perfect agreement. Converted into *p*-values significance was assumed at *p* < 0.05. Sensitivity is given as an average value in percent [19].

### Standard protocol approval and patient consent

All participants gave written informed consent. The study was approved by the local ethics committee (temporarily anonymized).

## Results

From 48 subjects screened for the study, 8 cases (7 in WMLs group, 1 in control group) had missing temporal bone windows and were excluded from further analysis. 29 patients in the WML group and 11 subjects from the control group, were considered for further US investigation and analysis. 29 patients showed vascular WMLs of variable degree quantified according to Fazekas grading scale (*n* = 13 I; *n* = 9 II; *n* = 7 III). Demographic and clinical data are depicted in Table 1.

Only images with reproducible clear anatomical landmarks per localization were included, therefore 233 localizations (161 with WMLs, 72 with normal MRI findings) were identified for further evaluation. In subjects with sufficient transtemporal bone window both ipsi- and contra-lateral localizations were analyzed.

TCS-MRI fusion imaging technique could visualize the MRI-verified WMLs in 147 of 161 localizations (sensitivity: 91%). The overall US detection rate of WMLs increased with higher degree of WML burden (I: 85%, II: 94%, III: 97%). The highest sensitivity was achieved at the contralateral central part (CPc) (97%), the contralateral dorsal horn (DHc) (96%) and the contralateral frontal horn (FHc) (95%). From the control group with normal MRI findings 72 localizations were identified for analysis. 54 localizations (specificity: 75%) were ultrasonographically identified consistently as normal.

The inter-rater analysis from 2 independent raters indicated an 86% agreement with an overall moderate strength of agreement (*κ*: 0.489, *p* < 0.0005) for all localizations. The highest accordance within raters was shown at the CPc; 92% (*κ*: 0. 645, *p *< 0.0005) and at the ipsilateral frontal horn (FHi); 90% (*κ*: 0. 644, *p *< 0.0005), both defined as substantial agreement; whereas, fair agreement was observed at the FHc, the CPi and the DHc. The detailed distribution of localizations and subjects is shown in Table 2. Quantitative analysis of degree of WMLs was not conducted based on US images.

**Table 2:** Detailed distribution of evaluated images per localization

WMLs	°I *(n* = 68)			°II (*n* = 54)			°III (*n *= 39)			Total WMLs (*n* = 161)		Normal (*n* = 72)			Total (*n* = 233)
Localizations (*n)*	*n*	UFI	Agr	*n*	UFI	Agr	*n*	UFI	Agr	UFI total (%)	Agr. total (%)	*n*	UFI	Agr	Kappa (*p*-value)
FHi (36)	13	9	8	14	13	12	9	8	7	30 (83%)	27 (90%)	18	18	15	*κ*: 0. 644 (*p* < 0.0005)
FHc (40)	19	17	14	10	10	10	11	11	9	38 (95%)	33 (87%)	14	5	4	*κ*: 0.370 (*p* < 0.0005)
CPi (15)	7	7	5	5	4	3	3	3	2	14 (93%)	10 (71%)	10	9	5	*κ*: 0.304 (*p* < 0.009)
CPc (37)	14	14	13	14	13	12	9	9	8	36 (97%)	33 (92%)	14	8	8	*κ*: 0. 645 (*p* < 0.0005)
DHi (5)	4	1	1	1	1	1	–	–	–	2 (40%)	2 (100%)	4	4	3	*κ*: 0.167 (*p* 0,386)
DHc (28)	11	10	8	10	10	8	7	7	6	27 (96%)	22 (81%)	12	10	6	*κ*: 0.268 (*p*: 0.003)
*n*	68	58	49	54	51	46	39	38	32	147	127	72	54	41	*κ*: 0.489 (*p* < 0.0005)
US detection and agreement in %		85%	72%		94%	85%		97%	82%	91%	86%		75%	76%	

The numbers (*n*) are given in total and in percent (%) of ultrasound (US) detection and inter-rater agreement.

*WMLs* white matter lesions, *Loc* localizations, *UFI* ultrasound fusion imaging, *Agr* agreement 2 raters, *FHi* frontal horn ipsilateral, *FHc* frontal horn contralateral, *CPi* central part ipsilateral, *CPc* central part contralateral, *DHi* dorsal horn ipsilateral, *DHc* contralateral dorsal horn

## Discussion

This study shows that transcranial B-mode sonography seems to be able qualitatively detect vascular WMLs based on MR images with a sensitivity of 91%. The combination of MR images with US images during US examination increases the detection rate of periventricular WMHs on US images in B-Mode since the MR images help to find the lesions with US. The highest sensitivity of WMHs ultrasound detection was shown at the contralateral central part and the contralateral frontal horn of the lateral ventricle and increases with WMH burden.

TCS is a well-established imaging technique in neurology to investigate brain parenchyma in various brain disorders and can complement information from other neuroimaging modalities [20, 21]. Brain CT or MR images superimposed to TCS insonation image show the almost perfect correspondence of anatomic structures intra- and extra-cranially and can aid the ultra-sonographer during the US session. Generally, the advantages of merging two imaging modalities for investigations or surgeries are common in other specialties (e.g., urology, neurosurgery) [22–24].

**Fig. 2:**
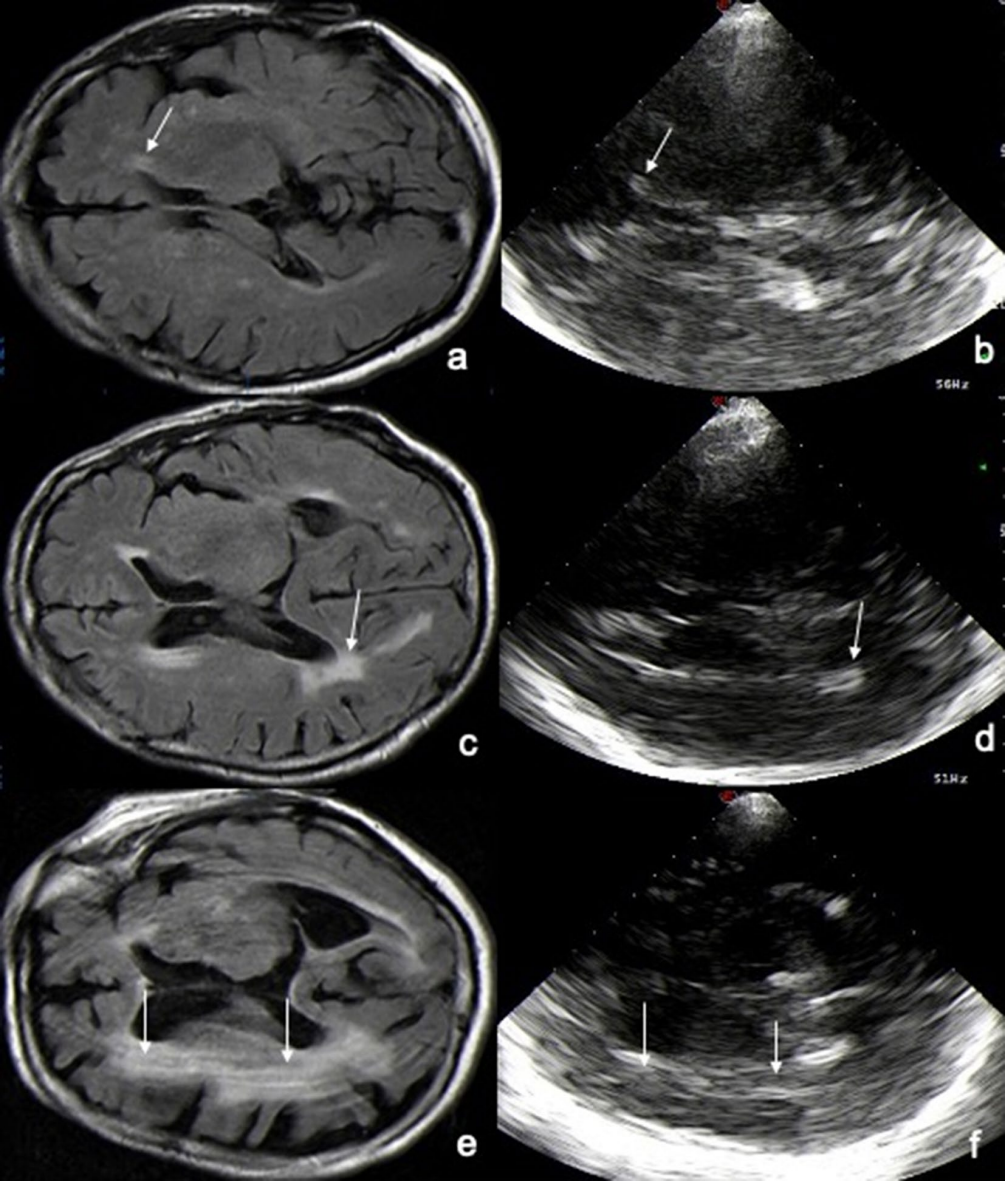
US visualization of WMLs based on MRI. The brain lesions seen on MR images correspond on ultrasound (US) images to hyperechogenic signals, double or blurred lines or as inhomogeneous hyperechogenic signal surrounding the wall of the central part, the frontal or dorsal horns of the lateral ventricles. Left: cerebral MR FLAIR image, axial plane. Right: transcranial sonography in B-Mode. **a**, **b**: WMLs I, white arrows indicate WML at the anterior horn ipsilateral. **c**, **d** WMLs II, white arrows indicate WML at the dorsal horn contralateral. **e**, **f** WMLs III, white arrows indicate WML in the deep white matter surrounding the central part contralateral

MRI-verified WMHs in periventricular location correspond to sonographic reproducible hyperechogenic structures, like double or blurred lines or hyperechogenic lesions surrounding the wall of lateral ventricles. In case of confluent deep white matter lesions, the brain parenchyma appeared more inhomogeneous in echogenicity with ultrasound in comparison to normal brain parenchyma, where the US image showed a more homogeneous echogenic pattern (Fig. 2). Maybe these change in echogenicity are more often seen on anatomical borderlines between brain parenchyma and lateral ventricle wall, and are more evident in case of WMH pathology. Especially in the control group sometimes we observed small hyperechogenic signals, like white caps, at frontal horns directly on the border of the ventricle wall and brain parenchyma, but thought as artifacts due to their inconstancy in appearance. This observation could be a reason for the low specificity.

Several US studies indicated that ionic deposits like iron, copper or manganese lead to hyper-echogenicity in different brain structures in neurodegenerative disorders [25–28]. Additionally, calcification can lead to an increased echogenicity. Petersen et al. recently reported a decrease of structural connectivity in patients with higher CSVD burden in which the frontal brain regions were prominently affected and hypothesized a disruptive effect on white matter fiber tracts [29]. Increased interstitial water content results in signal changes on MRI, typically on FLAIR and T2WI sequences [30]. Histologically in extensive WMLs decreased density of glial cells and vacuolization are the leading pathological findings, whereas subtle WMLs show a highly variable histopathology [31]. Conceivably, the loss of structural integrity, increased perivascular water content and damaged white matter fibers due to CSVD leading to a change of echogenicity with US. Further, transcranial Doppler sonography (TCD) data pointed out changes in the cerebral perfusion and vascular resistance, linked to microcirculation pathology and small vessel und capillary damage, in patients with VCI without evident dementia significantly associated with WMLs [32]. These changes might display the neurosonological correlates of microcirculatory pathology in patients with WMLs additionally to the TCS imaging findings. Contrary, some hemodynamic studies observed changes in TCD parameters in asymptomatic patients with Fabry´s disease, without any additional vascular risk factors or evident brain MRI lesions [33]. These findings have been considered to use TCD parameters as a screening method for patients at risk for both acute stroke and chronic cerebrovascular disease as well.

In newborn infants ultrasound examination of the brain is a common and reliable method. Correlation of US with MRI images in the detection of WMLs in newborns showed low reliability in subtle WMLs, while the visualization rate of severe and diffuse WMLs in the frontal and periventricular region with cranial US correlates well to hyperechogenic lesions identified on MRI [34–36]. These results are consistent with our observations. The highest sensitivity for WMLs US detection and inter-rater agreement was found in the frontal and central periventricular brain areas. At the posterior regions hyperintense artifacts were seen more often, mostly raising from the choroid plexus. UFI was a helpful tool to distinguish hyperechogenic artifacts from WMLs. Furthermore, the number of localizations at the CPi and the DHi is low, due to insufficient insonation angle and unreproducible depiction possibility of anatomical landmarks. Spotty lesions in the deep white matter were not visualizable with UFI as well.

The automatized quantification of echogenicity values from TCS-MRI fusion images in neurodegenerative disorders or of the insula demonstrates a high reliability using B-Mode Assist System [37, 38]. We evaluated US images without any automatized software, but constantly with the same presets. Further automatized imaging analysis should be tested to evaluate if the severity of WMLs burden could be graded with TCS.

This study has some limitations. US is known to be examiner dependent. Missing transtemporal bone window makes US examination impossible (in our study: WMLs group: 19%, control group: 8%). To apply fusion technique during TCS examination correctly, it is crucial to guarantee an almost perfect match to pre-acquired MRI images reproducibly. Therefore, standardized investigation protocols should be used. Due to anatomical variants the dorsal horn ipsi- and contra-laterally was not visualizable constantly in every patient. Also, the choroid plexus causes a hyperechogenic signal at the dorsal horn region, which sometimes makes it harder to visualize WLMs. In fact, the number in these localizations for evaluation is low. Further, the sample size is relatively low, because during the observation time of 3 months, there were no more patients who matched the inclusion criteria for this study.

Moreover, the question arises whether sonographically characteristic hints or patterns can be detected and defined to distinguish between vascular, inflammatory or degenerative white matter lesions with US, which sometimes is not fully differentiable on MR images alone. At this timepoint it is not clear if UFI/TCS is able to differentiate between vascular, demyelinating, metabolic cerebral WMLs. Further studies are necessary and planned to answer this question. The experimental insonation of white matter lesion in multiple sclerosis, performed in a few patients during study time, showed a different appearance on US images in comparison to vascular lesion. So, the hypothesis arises that this method maybe has the potential to differentiate these lesions etiologically.

## Conclusions

We described the US detection of periventricular vascular white matter hyperintensities in adults based on MRI lesions using B-Mode ultrasound fusion imaging technique; a further blinded study is planned. MRI is the valid gold standard imaging method in precise detection of WMLs. US could serve as an additional tool during routine TCS investigations as a screening method for CSVD. Incidental WLMs detection by routine TCS could then lead to early vascular risk evaluation in primary prevention.

## Data Availability

The dataset used and analyzed during the current study are available from the first author and the corresponding author on reasonable request.

## Abbreviations

CP(c)(i): Central part (contralateral) (ipsilateral)
CSVD: Cerebral small vessel disease
DH(c)(i): Dorsal horn (contralateral) (ipsilateral)
FH(c)(i): Frontal horn (contralateral) (ipsilateral)
IRA: Inter-rater analysis
K: Kappa
MCA: Middle cerebral artery
SD: Standard deviations
TCD: Transcranial Doppler sonography
TCS: Transcranial sonography
UFI: Ultrasound fusion imaging
US: Ultrasound
VCI: Vascular cognitive impairment
WMHs: White matter hyperintensities
WMLs: White matter lesions
